# Monitoring and Evaluation Indicators for Climate Change-Related Health Impacts, Risks, Adaptation, and Resilience

**DOI:** 10.3390/ijerph15091943

**Published:** 2018-09-06

**Authors:** Kristie L. Ebi, Christopher Boyer, Kathryn J. Bowen, Howard Frumkin, Jeremy Hess

**Affiliations:** 1 School of Public Health, University of Washington, Seattle, WA 98105, USA; cboyer10@uw.edu (C.B.); jjhess@uw.edu (J.H.); 2 University of Melbourne, Melbourne 3010, Australia; kathrynjbowen@gmail.com; 3 Institute for Advanced Sustainability Studies, 14467 Potsdam, Germany; 4 The Australian National University, Canberra 0200, Australia; 5 Our Planet, Our Health, Wellcome Trust, London NW1 2BE, UK; h.frumkin@wellcome.ac.uk

**Keywords:** adaptation, climate change, climate-sensitive health risks, indicators, resilience

## Abstract

Climate change poses a range of current and future health risks that health professionals need to understand, track, and manage. However, conventional monitoring and evaluation (M&E) as practiced in the health sector, including the use of indicators, does not adequately serve this purpose. Improved indicators are needed in three broad categories: (1) vulnerability and exposure to climate-related hazards; (2) current impacts and projected risks; and (3) adaptation processes and health system resilience. These indicators are needed at the population level and at the health systems level (including clinical care and public health). Selected indicators must be sensitive, valid, and useful. And they must account for uncertainties about the magnitude and pattern of climate change; the broad range of upstream drivers of climate-sensitive health outcomes; and the complexities of adaptation itself, including institutional learning and knowledge management to inform iterative risk management. Barriers and constraints to implementing such indicators must be addressed, and lessons learned need to be added to the evidence base. This paper describes an approach to climate and health indicators, including characteristics of the indicators, implementation, and research needs.

## 1. Introduction

Health adaptation indicators for monitoring, evaluation, and learning (M&E) are needed to track the health impacts of climate change, and efforts to adapt and build resilience. This tracking includes three main elements: (1) *vulnerability, risk, and exposure* for both populations and health systems; (2) *impacts* on population health and on health systems; and (3) *adaptation and resilience* in populations and health systems, across scales from local to national. M&E indicators should help identify good adaptation practices for replication and scaling up. The knowledge gained can inform iterative risk management of health systems, based upon learning, so that future adaptation actions are appropriately designed, refined, and implemented.

Standard health system M&E is not designed to track and foster health sector resilience, defined as “the capacity of health actors, institutions, and populations to prepare for and effectively respond to crises; maintain core functions when a crisis hits; and, informed by lessons learned during the crisis, reorganize if conditions require it.” [1]. In particular, current M&E approaches are not designed to incorporate or act upon information regarding significant environmental shifts or other hazards that have implications for operations and population health. The challenges that climate change adaptation present to standard health system M&E include:

- The need to recognize the role of weather, seasonality, climate variability, and long-term climate change on health outcomes and health sector operations.
- The need to consider decisions and their impacts over multiple overlapping time-scales.
- The need to acknowledge and address the inherent uncertainty in the rate, magnitude, and pattern of climate change for any one location [2].
- That climate change will have multiple, interacting downstream health effects, (e.g., on food and water security) and these one-to-many relationships complicate the development of specific indicators.
- That future climate change trajectories depend on current mitigation choices, making it difficult to determine how to bound indicators temporally.
- That adaptation decisions in health and other sectors will have significant impacts on the burden of climate-sensitive health outcomes and on health sector operations, and the many-to-one relationship can make it difficult to identify indicators of principal relevance to health.
- Due to inherent uncertainties and long-term trends, a significant element of climate change adaptation relates to institutional learning and knowledge management to facilitate iterative risk management, in which information regarding changing hazards associated with climate change is continuously integrated into processes that prepare for and manage health risks over time [3,4,5].

Given this wide range of challenges, standard indicators for climate-sensitive health outcomes will not adequately capture the processes of changing risks, adaptation effectiveness, and resilience. In addition to monitoring the health impacts of climate change, indicators of vulnerability and exposure, health system resilience, and learning and knowledge management are needed. Indicators should reflect not only outputs such as early warning systems [6], but also system changes that ensure longer-term resilience. Examples include institutional agreements to support data sharing and analysis, professional development strategies enabling staff to integrate new data streams and models into adaptation planning, and commitments to maintain sufficient human and financial resources—all of which can be tracked. Accordingly, indicators of institutional engagement [7] should describe key steps in the process of adaptation, particularly the extent to which capacity is being built for institutional learning and iterative management approaches that promote resilience across a range of possible climate and development futures [8].

Health systems are increasingly focused on “evidence-based public health” that develops, implements, and evaluates the effectiveness of programs and policies [9]. Evidence of the magnitude and pattern of public health problems and interventions to manage them are systematically assembled, evaluated, and integrated into decision-making [10]. The emphasis is on synthesizing, applying, and disseminating knowledge (e.g., knowledge translation). Evidence-based public health efforts dovetail with those of implementation science, which is focused on the dissemination of effective interventions and practices [11]. With modifications to account for the differences between climate change and other exposures, following an evidence-based public health approach and applying the principles and approaches of implementation science would support climate change adaptation by identifying the most effective interventions to protect health and well-being in a changing climate, supplementing current approaches [10]. Many of these steps can be used to develop indicators.

Some relevant indicators sit outside of the health sector. Monitoring climate and health and evaluating health system resilience does not occur solely within health systems. Indicators of exposure, for example, are monitored by weather and climate services, and some indicators of vulnerability, such as socioeconomic or geographic vulnerability, are typically measured by other government departments. Some indicators of system resilience, particularly those related to infrastructure, are monitored by other sectors, such as electrical power, while other relevant indicators related to risk distribution, (e.g., insurance coverage) are monitored by the public and insurance sectors. While the health sector is not responsible for developing or monitoring these indicators, they are relevant to core service provision and should be a part of a comprehensive package of climate-related indicators.

## 2. Methods

In this paper, we review major efforts to identify indicators relevant to climate change and health. Based on this review and extensive experience with health adaptation, we discuss three categories of indicators that should be considered for more comprehensive climate change and health M&E programs: (1) indicators of health vulnerability, exposure, and risk related to climate variability and change; (2) indicators of climate change impacts on population health and health systems; and (3) indicators of adaptation processes and of health system resilience, including coordination and collaboration across scales and with other sectors. We then discuss constraints on indicator development and application, and explore application of the principles discussed through the example of indicator development for the Ministry of Health in Cambodia.

## 3. Review of Indicators of the Health Risks of and Adaptation to Climate Change

Developing indicators of the burden of climate-sensitive health outcomes began relatively recently (c.f. [12]); [13]. Indicator sets have been proposed by health ministries, multilateral organizations, and academic coalitions. 

In the United States, two early efforts were led by the Council of State and Territorial Epidemiologists (CSTE) and the U.S. Environmental Protection Agency (EPA) respectively. The CSTE effort yielded a list of indicators selected for completeness, usability, and accuracy, measuring environmental and health parameters, vulnerability, and both mitigation and adaptation actions [12]. Notably, few available data sources were identified to populate the latter indicators. The EPA proposed a similar set of indicators based on surveillance data and programs. Some indicators focused on exposure, such as temperature extremes and ragweed pollen season length, and others on health outcomes, including incident cases of heat-related deaths, heat-related illnesses, Lyme disease, and West Nile virus infection (https://www.epa.gov/climate-indicators) [14]. These indicators are a fraction of the wide range of climate-sensitive health outcomes of concern but were considered a sensible start based on available data.

In Canada, Cheng and Berry [13] evaluated 77 climate change and health outcome indicators based on their specificity, availability, feasibility, quality, comparability over time and place, and relevance to planning. Eight indicators scored high enough to be included in the final suite of indicators focused on the burden of climate-sensitive health outcomes: excess daily all-cause mortality due to heat (modeled); premature deaths due to ozone and particulate matter (PM_2.5_) (modeled); preventable deaths from climate change (modeled); disability-adjusted life years lost from climate change (modeled); daily all-cause mortality (trends associated with heat and air pollution); daily non-accidental mortality (trends associated with heat and air pollution); West Nile disease incidence in humans; and *Lyme borreliosis* incidence in humans.

The Lancet Commission on Health and Climate Change proposed an ongoing tracking process involving indicators [15,16], including such parameters as exposure to temperature change, exposure to heat waves, changes in labor productivity, exposure to floods, exposure to drought, changes in the incidence and geographic range of climate-sensitive infectious diseases, and food security and under-nutrition. This gave rise to the Lancet Countdown on Health and Climate Change, which published its first annual report in 2017 [16]. The Countdown monitors an evolving set of indicators related to health impacts, health system preparedness, mitigation and adaptation activity, finance and policy. The Countdown is accompanied by country-specific reports that explore similar trends at a national level for selected countries (http://www.lancetcountdown.org/the-report/). The indicators reported in the Countdown are useful for characterizing global trends and supplement national and local indicators.

The World Health Organization proposed a wide range of indicators to monitor the extent to which a health system is able to anticipate, respond to, cope with, recover from, and adapt to climate-related shocks and stress, so as to bring sustained improvements in population health, despite an unstable climate [17].

Every context is different, and there is no standard and universal set of indicators related to climate and health, just as there is no one-size-fits-all M&E program for climate change and health adaptation. Indicators should be developed based on the needs and level of capacity of national to local entities, recognizing that in some instances, it may be possible to develop common indicators that allow for comparison across settings.

## 4. Three Categories of Indicators to Protect Health from Climate Change

### 4.1. Indicators of Vulnerability and Exposure to Climate Variability and Change

Population health vulnerability is the summation of all risk and protective factors that determine whether an individual or subpopulation experiences adverse health outcomes from exposure to a climate-related hazard [18]. Vulnerability indicators are designed to assist health officials and others to identify populations that are at particular risk for adverse health outcomes because of climate change. Indicators of vulnerability to the health risks of climate change are, in many instances, already being collected. Examples include the numbers of those living in poverty, the numbers of children and pregnant women, the numbers with chronic diseases that increase susceptibility to adverse climate-sensitive health outcomes, and the number of people/communities exposed to extreme weather and climate events (e.g., floods and drought).

In addition, countries collect data on access to health care services, the status of the public health and health care delivery infrastructure, access to and quality of education, availability of resources, health insurance coverage, and other social determinants of health that influence vulnerability [19]. In addition, indicators of relative wealth or poverty and income inequality provide information on socioeconomic factors that can interact with climate-related hazards in determining sensitivity to climate variability and change. Geographic indicators of increased risks for specific climate-sensitive health outcomes due to, for example, the baseline climate or location, provide additional information on vulnerability. In health systems, factors influencing vulnerability include the ability of healthcare facilities to manage an extreme event. Additionally, data are collected on the extent to which communities and regions are exposed to climate-related hazards, such as heatwaves, flooding, and drought. Effectively monitoring vulnerability calls for developing integrated programs that provide information needed by a range of sectors, at useful geographic and temporal scales based on the risks that need to be managed.

### 4.2. Indicators of the Health Impacts of Climate Change

Most proposed indicators for climate change and health M&E focus on measuring the impact of climate change on population health and health systems. Data are likely to be available in many jurisdictions for:

- Excess mortality associated with exposure to high ambient temperatures;
- All-cause and cause-specific morbidity and mortality associated with other extreme weather events;
- Respiratory disease mortality from exposure to air pollutants such as ozone and particulate matter;
- Changes in the incidence and geographic range of climate-sensitive infectious diseases, with the specific diseases chosen varying depending on which are important or expected to be important in a country or region; and
- Undernutrition (generally measured as stunting).

In some countries, it may be possible to model an aggregate measure of the overall burden of disease that could be attributed to a changing climate, such as disability adjusted life years (DALYs) or years of life lost (or a comparable metric) from climate variability and climate change, respectively. Other indicators can be added, depending on the local or regional climate-related exposures that can cause adverse health outcomes, such as injuries, illnesses, and deaths attributed to wildfires, or the numbers of asthmatic episodes associated with high pollen events.

The purpose is not to just track these indicators but to analyze patterns of change over time. Useful analyses might focus on absolute and relative changes in population exposure, in climate-sensitive health outcomes, and on the association between particular exposures and health outcomes. For example, while exposure to heatwaves may increase over time, the rate of adverse health outcomes should decrease with effective interventions and increased awareness.

There are multiple sources of surveillance data for tracking these indicators. The World Health Organization (http://www.who.int/healthinfo/statistics/en/), the World Bank (http://datatopics.worldbank.org/ hnp/) and the University of Washington Institute for Health Metrics and Evaluation (IHME) (http://www.healthdata.org/), among others, provide country-level data on deaths, DALYs, and life expectancy. Through IHME, for example, data are available at the national level from 1990 onwards for 249 causes of death. These data could be used to quantify changes in the burden of disease over time.

Surveillance programs need to be augmented to collect data to support modeling of future projections of and adaptation options to effectively prepare for risks over the coming decades. This includes monitoring humans, vectors, and disease pathogens in areas where climate change could facilitate vector-borne diseases changing their geographic range.

Indicators for monitoring adverse health outcomes also should align with the indicators established for monitoring the Sustainable Development Goals (SDGs), the Sendai Framework for Disaster Risk Reduction, and other relevant global frameworks, to facilitate implementation strategies, effective resource allocation, and measurement of progress [20].

### 4.3. Indicators of Adaptation and Health System Resilience

#### 4.3.1. Effectiveness of the Process of Adaptation

Health adaptation aims to reduce the burden of climate-sensitive health outcomes. Accordingly, indicators of adaptation effectiveness might track changes in the geographic range, seasonality, and incidence of climate-sensitive outcomes. Since adaptation occurs within the context of changing state and national development patterns, indicators should also track adaptive capacity—the capacity of individuals, communities, and health systems to manage increases in the frequency and intensity of extreme weather and climate events and changing burdens of climate-sensitive health outcomes.

In a larger sense, adaptation also entails a commitment to and management of institutional learning processes. Thus, indicators should measure adaptation as an outcome (e.g., adapted to a risk, with reduced rates of the outcome as a result) and as a process (e.g., political and institutional commitment to adaptation engagement and the presence of systems and processes for facilitating institutional learning).

Process indicators are needed to monitor the extent to which sufficient human and financial resources are available to support adaptation programs and projects because plans without budgetary allocations are unlikely to be implemented or sustainable. Many low-income countries face the issue of absorptive capacity, indicating that even if there is sufficient financial support for activities, there may not be sufficient human capacity for implementation.

Outcome indicators are suitable for evaluation and can provide strong indications of whether adaptation goals are being met. Outcome indicators also provide information that is useful for assessing system performance, particularly when compared with a counterfactual expressing an adaptation goal. For instance, process indicators may track implementation of an extreme heat early warning system, while outcome indicators related to all-cause mortality rates can help determine how well the early warning system is protecting health during periods of extreme heat exposure.

There is limited consensus on the criteria for determining whether an adaptation program or project is a success, with evaluations taking different approaches [21]. Indicators of success are typically observable, concrete process measures describing program implementation. Examples include indicators of the extent and effectiveness of plans incorporating climate resilience measures in water safety plans, infectious disease control programs, etc.; and indicators of the effectiveness of measures implemented to manage climate-sensitive health outcomes, including the success (or not) of approaches to adaptive management, the extent to which adaptive capacity is being built based on the number of people trained following a project, and other related issues.

Other indicators should measure the capacity of health systems to prepare for and manage the risks of climate change over time [22], addressing the awareness of longer-term climatic trends, organizational diversity, internal monitoring, and learning management to facilitate self-regulation and vertical and horizontal integration (with other health system levels and other sectors). Examples include monitoring the frequency with which vulnerability and adaptation assessments are updated, and tracking progress on integrating health into National Adaptation Plans through the Paris Agreement and Nationally Determined Contributions and the extent to which they are implemented. Indicators also could measure the awareness of the health risks of climate change, as measured by the number of general practitioners and other health personnel trained in climate change; and the extent of public awareness of and actions to address the health risks of climate variability and change. Other relevant indicators include pathways established for integrating and regularly updating environmental information, the presence of knowledge management systems and their application in building resilience, and indicators of coordination across the health system and with other sectors that provide services essential to health system function.

Effective adaptation requires active engagement of health systems with other ministries and organizations. This engagement must recognize that vulnerabilities and capacities in all sectors change over time, and that systems-based approaches are needed to facilitate adaptation across geographic scales and administrative units. Examples of possible indicators include: (1) the existence and effectiveness of collaborative mechanisms (e.g. memoranda of understanding) with other departments and ministries, such as meteorological services, to measure the extent these organizations are sharing data and coordinating efforts to manage risks that span sectors; and (2) the extent of local to national government commitments to climate change adaptation, such as by incorporating adaptation strategies into development plans and budgets. Social network analysis [23] can be used to measure the extent of coordination and collaboration across organizations and institutions.

In addition, local to national indicators (and means of verification) are needed that measure the extent to which public health and health care policies and programs:

- Assess and manage climate-related risks from a systems perspective, taking into consideration the multiple environmental and social drivers of the geographical range, seasonality and incidence of health outcomes;
- Design, implement, monitor and evaluate interventions using projections of health impacts under different climate and socioeconomic futures; and
- Explicitly incorporate learning (informed by monitoring and evaluation) into iterative management cycles, building capacity and managing knowledge for further adaptation as the climate continues to change.

#### 4.3.2. Building Health System Resilience

The health sector has come relatively late to the concept of resilience as a guiding principle. Preconditions for resilience in the health sector include understanding of the global scope of potential health system crises, clear awareness and consensus regarding the roles and responsibilities of actors at different levels of the global health system, a legal and policy foundation to facilitate action and ensure accountability, and a strong and committed health workforce; all are currently lacking [1]. A resilient health system is aware, diverse, self-regulating, integrated, and adaptive [1].

Ideally, adaptation enhances system resilience. However, indicators for tracking the adaptation process may provide limited insights into the extent to which a system will exhibit longer-term resilience. 

Linking indicators across local to national scales can provide a more complete and nuanced understanding of where a community or a nation stands in the process of adaptation, progress that has been achieved, and additional efforts that could be helpful.

## 5. Constraints to Developing and Implementing Indicators

A wide range of constraints and barriers to health sector adaptation present multiple challenges to developing and implementing indicators. An inherent uncertainty is what indicators will be needed at what points in time as the climate continues to change and health risks emerge [24]; for instance, indicators related to sea level rise and related migration will become relevant at different points in time. Better understanding is needed of the multiple drivers of adverse climate-sensitive health outcomes and how they could interact with climate change and development scenarios in ways that could alter risks over time. Understanding is also needed of how the multiple upstream drivers of adverse health outcomes could interact in ways that could alter health burdens and adaptation effectiveness. For example, the top five upstream drivers of infectious disease threats in Europe are (in order of importance) travel and tourism, food and water quality, natural environment, global trade, and climate [25]. This suggests that indicators in health systems need to be linked with indicators in other sectors to ensure information is collected to support efforts to prevent possible future disease outbreaks, including addressing mismatches of datasets and ownership issues.

Developing, monitoring, and evaluating indicators of risks and effectiveness of adaptation options requires human and financial resources. Although there is widespread agreement of the importance of M&E within health systems, the extent to which expertise and financial resources are available is highly variable. Further, developing and maintaining the requisite datasets requires investments in surveillance and monitoring programs, and in capacity building in resource constrained situations to implement and maintain these programs and associated analyses [26]. It would be useful to prioritize projects that address urgent and immediate needs or that provide multiple benefits (e.g., win-win). Another key constraint is information needed for M&E, including:

- Data to develop robust baselines against which to measure changes in the burden of climate-sensitive health outcomes and of the effectiveness of adaptation. Such data are limited in many low- and middle-income countries, and in low resource settings in high-income countries.
- Data at finer temporal scales than at the scale of national or large sub-national regions, including the distribution of vulnerable groups within regions. Data on the number of cases of reportable health outcomes are available at national and large sub-regions within countries but may not be available for smaller geographic regions.
- Data on health risks of climate change, such as mental health, whose risks may be under-represented.
- Data collected using uniform definitions and methods to develop comparable indicators. Outside of the International Health Regulations (http://www.who.int/topics/international_health_regulations/en/), definitions and methods for collecting health data vary.

## 6. Example: Developing Climate Change and Health M&E Indicators for the Cambodian Ministry of Health

Indicators need to be grounded in the national context. An example of developing nationally relevant health indicators is from the Royal Government of Cambodia (RGC). The RGC, with support from various organizations and agencies, established an overall national M&E framework for climate change adaptation, including a framework for health. Major priorities for health adaptation identified by the Ministry of Health’s National Climate Change Action Plan for Public Health include vector- and water-borne diseases and the health impacts of extreme weather events. M&E indicators are needed to guide programming and policy development, establish early-warning systems, increase public awareness, and develop assistance programs, within the context of limited resources and capacity, as well as high vulnerability.

With support from WHO and based on consultation meetings with stakeholders, a tool was developed to guide the Ministry of Health (MoH) in selecting indicators for assessing the effectiveness of health adaptation to climate change (MoH, personal communication December 2017). The process is institutionally rooted in the MoH to promote ownership, sustainability, and capacity building by continuously tracking long-term trends in the risks of climate change for health. This aligns with mainstreaming efforts proposed by international agencies [27]. Categories of M&E indicators were developed based on the literature, earlier frameworks, and stakeholder consultation: health vulnerability and exposure; burden of climate sensitive health outcomes; health adaptation and resilience; coordination and collaboration. Specific indicators build off and update current frameworks and, when possible, utilize existing and routinely collected data, or data that are or could be collected. These indicators aim to fit into the over-arching framework for monitoring and evaluating of climate change adaptation in Cambodia, as well as broader national and international sustainable development goals. Lastly, the tool maintains flexibility so that it will prove useful for future health adaptation projects [28].

## 7. Research Needs

M&E for adaptation will be increasingly called upon to describe the effectiveness of current efforts and to estimate the adaptation gap that will need to be filled to promote resilience within the context of a changing climate, which means the research needs are significant. Research is needed regarding the most appropriate and effective indicators among those discussed above as well as their use, negotiation of barriers and constraints, and dissemination of best practices across health systems. Indicators of the health risks of and adaptation to climate change should feature (i) sensitivity (i.e., the extent to which the metric accurately detects changes in health status or institutional performance), (ii) construct validity (i.e., the extent to which the metric measures what is intended), and (iii) usefulness (i.e., metrics are practical and easily understood by public health practitioners, decision-makers, and other personnel). This research should build on and complement the international and national efforts on indicator development and refinement summarized in Section 3, including providing insights into new indicators at all geographic scales.

There are several areas where additional research could provide useful insights for developing local to national measures of how effectively health sector policies and programs increase resilience to climate change and for communications regarding those indicators:

(1) Assessing and prioritizing the health risks of climate change over spatial and temporal scales. Health systems traditionally prioritize surveillance and monitoring based on either the current burdens of disease or the potential for infectious diseases to cause epidemics. Climate change will likely affect both, with changes in the magnitude and pattern of climate-sensitive health outcomes as the climate continues to change. Proactive prioritization using environmental information (e.g., projected changes in temperature and precipitation) could prevent additional morbidity and mortality. These indicators would relate to processes, (e.g. utilization of environmental information) as well as outcomes, (e.g. early warning of a vector-borne disease outbreak).
(2) Assessing and managing risk from a systems perspective, taking into consideration the multiple environmental and social drivers of the geographic range, seasonality, and incidence of health outcomes. More research is needed to understand the best way to use surveillance data to identify disease thresholds, interface with emergency preparedness, and project future burdens. For example, indicators are needed to monitor the robustness of health surveillance systems as climate change-related health threats emerge and intensify in some regions, including indicators of emergency preparedness to better protect population health. Research is needed to identify key environmental variables to include in surveillance systems linked to health outcome data or to establish proxy data (e.g., pollen, harmful algal blooms). Further, long-term studies are needed to help quantify the relationships between meteorological variables and health outcomes, and allow for evaluation of shifting distributions of vectors and pathogens across time.
(3) Use of projections of climate change health impacts under different climate and socioeconomic futures to design and implement interventions, to direct disease surveillance strategies, to make choices regarding early warning systems, and to develop other programs to avoid, prepare for, and cope with the changes and new threats expected to arise. This includes identifying how indicators can be used to identify disease thresholds in different geographic regions. This may be most effectively achieved through co-design of indicators with stakeholders [29].
(4) Explicitly incorporate learning (informed by M&E) and knowledge management (i.e., integration of new environmental and other data streams) into iterative management cycles and use of models for decision making, as well as building capacity for further adaptation as the climate continues to change;
(5) How to most effectively communicate indicators to engage the public and provide information regarding climate change preparedness and protections against climate-sensitive hazards; and
(6) How to communicate indicators to motivate and inform policy decisions, prioritize ongoing investments in health protection related to climate change, and characterize returns on health protection investments at different time horizons in a changing climate.

Of note, many of these themes relate to the priority of increasing resilience to other threats to the global health system such as pandemic disease, and the research proposed here can facilitate adaptation and resilience to a wide range of hazards.

## 8. Discussion

Evidence of adverse health impacts attributable to climate change on health is growing [30] and further increases in climate-sensitive disease burden are projected, some of which can be reduced with additional adaptation policies and programs [24]. These trends underscore the urgent need for a suite of indicators to monitor and evaluate the ability of communities and nations, and their health systems, to prepare for and effectively manage the health risks of a changing climate. The uncertainties and complexities of climate change and of future development choices mean that any set of indicators need to be flexible, providing a strong element of learning to inform institutional learning and knowledge management. Established M&E indicators for health systems need to be modified to provide the information needed to track the effectiveness of adaptation.

Despite being oft repeated, adaptation is not only (or sometimes even primarily) local. Whilst most climate change impacts are indeed experienced locally, such as floods, reductions in crop yields, or spread of disease, these localized impacts can have national and international ramifications that require action beyond the local level. For example, the heatwaves and fires in and around Moscow in 2010 affected wheat exports that had consequences for other countries, including impacts on food security [31]. The growing risks from climate variability and change, and increasing interest from development partners, mean that many local actors are implementing adaption options designed by and for local human and natural systems, such as mangrove restoration to reduce vulnerability to storm surges. Ideally, these should be embedded within national adaptation and development plans, to ensure the adaptation options implemented directly or indirectly promote achieving national development objectives, including the protection of human health and well-being.

At the same time, national policies and institutions can affect the magnitude and pattern of impacts by affecting local vulnerability and the capacity to respond. National priorities, constrained national human and financial resources, and other factors influence the extent to which a nation focuses on addressing poor and underserved regions most likely to be affected by climate variability and change. Choices made on locations of critical infrastructure, for example, have historically been made without consideration of the potential consequences of increases in the intensity of extreme weather and climate events for human health and well-being. Further, international donors can influence national development priorities, which can have consequences for local vulnerability.

Preparing for the significant challenges of climate change means developing new technical knowledge and capacities, enhancing current and developing novel surveillance, and facilitating engagement across sectors where climate change-related impacts may affect human health. Efficient indicators can track progress made, and highlight when and where midcourse corrections would increase the effectiveness of adaptation programming. Rising to these challenges will support the next transformation of public health [32], to ensure robust health systems continue to protect and promote population health in the 21st century.
